# Genome Wide Identification of Aberrant Alternative Splicing Events in Myotonic Dystrophy Type 2

**DOI:** 10.1371/journal.pone.0093983

**Published:** 2014-04-10

**Authors:** Alessandra Perfetti, Simona Greco, Pasquale Fasanaro, Enrico Bugiardini, Rosanna Cardani, Jose M. Garcia. Manteiga, Michela Riba, Davide Cittaro, Elia Stupka, Giovanni Meola, Fabio Martelli

**Affiliations:** 1 Molecular Cardiology Laboratory, IRCCS-Policlinico San Donato, San Donato Milanese, Milan, Italy; 2 Epigenetics & Regenerative Pharmacology, IRCCS Fondazione Santa Lucia, Rome, Italy; 3 Department of Neurology, University of Milan, IRCCS-Policlinico San Donato, Milan, Italy; 4 Center for Translational Genomics and Bioinformatics, San Raffaele Scientific Institute, Milan, Italy; 5 Laboratory of Muscle Histopathology and Molecular Biology, IRCCS-Policlinico San Donato, San Donato Milanese, Milan, Italy; University of California, Los Angeles, United States of America

## Abstract

Myotonic dystrophy type 2 (DM2) is a genetic, autosomal dominant disease due to expansion of tetraplet (CCTG) repetitions in the first intron of the ZNF9/CNBP gene. DM2 is a multisystemic disorder affecting the skeletal muscle, the heart, the eye and the endocrine system. According to the proposed pathological mechanism, the expanded tetraplets have an RNA toxic effect, disrupting the splicing of many mRNAs. Thus, the identification of aberrantly spliced transcripts is instrumental for our understanding of the molecular mechanisms underpinning the disease. The aim of this study was the identification of new aberrant alternative splicing events in DM2 patients. By genome wide analysis of 10 DM2 patients and 10 controls (CTR), we identified 273 alternative spliced exons in 218 genes. While many aberrant splicing events were already identified in the past, most were new. A subset of these events was validated by qPCR assays in 19 DM2 and 15 CTR subjects. To gain insight into the molecular pathways involving the identified aberrantly spliced genes, we performed a bioinformatics analysis with Ingenuity system. This analysis indicated a deregulation of development, cell survival, metabolism, calcium signaling and contractility. In conclusion, our genome wide analysis provided a database of aberrant splicing events in the skeletal muscle of DM2 patients. The affected genes are involved in numerous pathways and networks important for muscle physio-pathology, suggesting that the identified variants may contribute to DM2 pathogenesis.

## Introduction

Myotonic dystrophies are dominantly inherited multisystemic disorders characterized by muscle weakness, myotonia, CNS involvement and cataracts.

Two types of DM have been described. Myotonic dystrophy type 1 (DM1) or Steinert’s disease (DM1, OMIM 160900) is one of the most common forms of muscular dystrophy in adults with a prevalence of 1/8000 worldwide [1]. It is caused by an expanded (CTG)_n_ repeat in the 3’ untranslated region of the *Dystrophia Myotonica Protein Kinase* (DMPK) gene [2]–[4].

Myotonic dystrophy type 2 (DM2, OMIM 602688) displays a prevalently proximal impairment and milder clinical symptoms than DM1. It is caused by the expansion of a tetranucleotidic repetition (CCTG)_n_ in the first intron of the *CCHC-type zinc finger, nucleic acid binding protein* (CNBP) gene [5].

The disease mechanism proposed for both DM types involves a toxic gain-of-function by RNA: the mutation causes the accumulation of the expanded CUG/CCUG transcripts into nuclear RNA foci [6], which sequester RNA-binding proteins, such as MBNL1 (Muscleblind-like 1) that decreases its activity in DM patients [7], while the amount of CELF1 (CUGBP/Elav-like family member 1) increases [8]–[10]. These events lead to the expression of aberrant embryonic protein isoforms in adult tissues, muscular as well as other [11]. Thus, the pervasive spliceopathy observed in DM is indicated as a likely cause of the multisystemic features of this disease [12].

Alternative splicing (AS) is a post-transcriptional event whereby exons are joined by different combinations generating various isoforms from a single gene. It has been shown that most genes have at least 2 alternative isoforms [13], [14]. The expression of AS-generated isoforms can be tissue-, development- or sex-specific and these isoforms can fulfill different or even opposing functions [14]–[16]. Several studies investigated a limited number of genes aberrantly spliced in DM1, DM2 or both [17]–[26], but so far, only few studies investigated genome-wide changes in splicing events in DM1 and DM2 patients: one was limited to muscle specific genes [21]; another recent investigation profiled both DM1 and DM2 patients, but the validation phase was restricted to DM1 patients [27] and a third one admittedly displayed relatively low sensitivity [28]. Given the high variability of DM patients and the limited number of patients that is feasible to recruit, due to the rarity of the disease, it is clear that more insight on the AS aberrations in DM2 skeletal muscles is needed.

The GeneChip Human Exon 1.0 ST Array investigates the expression of virtually all known and many predicted human exons (∼1 million) allowing genome-wide evaluation of splicing events. Therefore, we used the exon array technology to explore DM2-related alternative splicing in muscle biopsies. We identified more than 200 genes displaying aberrant AS in DM2, likely affecting DM2-deregulated pathways and networks.

## Materials and Methods

### Patients selection and skeletal muscle biopsies

This study was authorized by the Institutional Ethics Committee (Azienda Sanitaria Locale-Milano 2) and was conducted according to the principles expressed in the Declaration of Helsinki, the institutional regulations and Italian laws and guidelines. All biopsy specimens were taken after specific written informed consent was obtained. Human muscle biopsies from *biceps brachii* were harvested under sterile conditions and snap-frozen in liquid nitrogen. Clinical diagnosis of DM2 was based upon the criteria set by the International Consortium for Myotonic Dystrophies [29]. Fluorescence *in situ* hybridization was performed on frozen muscle sections to confirm DM2 diagnosis according to Cardani et al. [30]. Control (CTR) biopsies were from subjects admitted with suspected neuromuscular disorder of undetermined nature. CTR biopsies did not show overt signs of muscle pathology upon on histological and immunohistochemical examination. All muscle biopsies were processed by the same pathology team and each was analyzed by two expert pathologists.

### Sample preparation and data analysis

**Figure 1:**
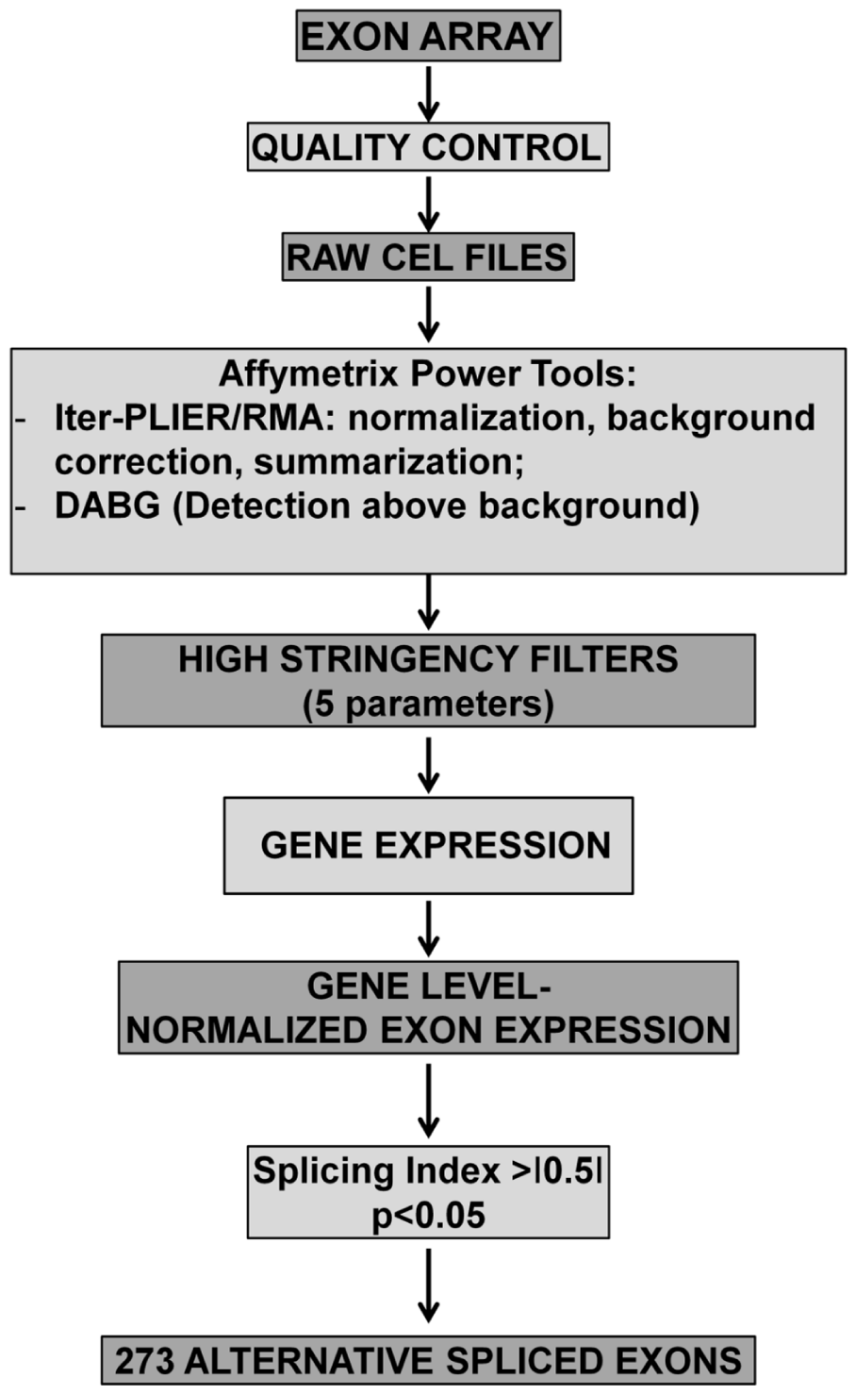
Array analysis workflow for AS identification. The scanning of hybridized GeneChip Human Exon 1.0 ST Affymetrix microarrays, after quality control, generated raw CEL files. These were uploaded on Exon Array Analyzer and processed by Affymetrix Power Tools (APT). Iter-PLIER and RMA performed a data pre-processing, while DABG calculated *p*-values to indicate if the exon signal was detected within or above the background noise. Next gene expression, gene level normalized exon expression, splicing index were calculated. Only events with a p value<0.05 and a splicing index <-0.5 or >+0.5 were considered. Next, 5 further selection criteria were applied in order to minimize the number of false positives (see Methods). This analysis yielded 273 alternative spliced exons in DM2 patients.

Total RNA was extracted using TRIzol (Invitrogen) and the TissueLyser system (Qiagen) and gene expression profiles were measured using the Gene Chip Human Exon 1.0 ST Array (Affymetrix) as previously described [31]. CEL files (GEO dataset GSE37794) with raw data were uploaded to the Exon Array Analyzer (EAA) 2.2 server (http://eaa.mpi-bn.mpg.de/) [32]. EAA first executed the Affymetrix Power Tools (APT) (http://www.affymetrix.com) for background correction, normalization and summarization of raw signals (Fig.1). These software implemented Iter-PLIER and RMA (Affymetrix Inc., 2005) for exon and gene-level processing, respectively, as well as the Detection Above Background (DABG) method. Exon-level data were filtered to include only those probe sets that were included within the ‘‘Core Meta-Probeset’’, composed by 17,800 transcript clusters of RefSeq and full-length GenBank mRNAs (Release March 2006-NCBI36/hg18). To reduce false positives, the following transcripts/probe sets were discarded: 1) probe sets that were not expressed in at least one group (DABG p-value >0.05 in >50% of the samples); 2) transcript clusters that were not expressed in both groups (>50% of their probe sets not detected in >50% of samples); 3) probe sets with high potential for cross hybridization, i.e. probe sets with only one hybridization probe positive; 4) genes with very large expression differences (≥10-fold difference); 5) probe sets with very large gene-level normalized intensities (≥5) [32], [33] (http://eaa.mpi-bn.mpg.de/; http://www.affymetrix.com/support/technical/technotes/id_altsplicingevents_technote.pdf).

Gene-level normalized intensities (NI) and the Splice Index (SI) [34] were calculated as indicated below. 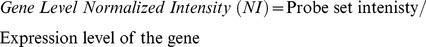


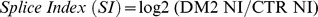



The R/Bioconductor [35], package LIMMA [36] was used for differential analysis; the Student’s t-test of gene-level normalized intensities was calculated and corrected for multiple comparison [37]. The Splice Index, gene level normalized intensities and p-values were imported into MySQL tables, which were used by the web interface to display results and generate graphics.

### Reverse transcription PCR validation

mRNA levels were measured using the SYBR-GREEN quantitative PCR (qPCR) method (Life Technologies) as previously reported [31] using a 7900HT Fast Real Time PCR System (Applied Biosystems). cDNAs were amplified using specific primers indicated in Table S1; data results were normalized against RPL13 and relative expression was calculated using the comparative Ct method 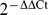 [38]. Each sample was measured in triplicate and values were averaged. For the CaMK2G gene, reverse transcription PCR products were also resolved onto a 5% polyacrylamide electrophoresis gel, stained with ethidium bromide for UV visualization, and the extracted bands, which represent the two different isoforms, were verified by DNA sequencing.

### Bioinformatic analysis of pathways and functions

Pathway analysis was performed using Ingenuity Pathways Knowledge Base-v8.8 (Ingenuity Systems, content version 17199142, release date 17/09/2013) using Genes with LIMMA adjusted p-value <0.05 and -0.5>S.I.>0.5 as reference set and assuming direct and indirect relationships. Fisher’s exact test p-value<0.05 was deemed as statistically significant.

For network analysis, first, we built Metacore Networks (Analyze Networks algorithm, v6.13 build 43450, GeneGO, Thomson Reuters) using DMPK gene and Myotonic Dystrophy related genes as inputs for the algorithm (ACE, ATXN1, CDC42BPB, CNBP, CELF1, CAPNS1, DMPK, DMWD, DMD, INSR, CDC42BPA, FXYD1, TNFRSF1B, TNF, TSPAN7, TNF, TNFRSF1B) and looked for genes in our dataset that intersected the top-score sub-networks related to DM. Next, we created a core network of direct interactions amongst our LIMMA significant genes; we also expanded the network by adding genes outside our dataset, whose interaction is curated in MetaCore. We identified smaller network communities that were prioritized by number of genes in our dataset. We used this second approach to analyze our data set without any prior knowledge.

### Statistical analysis

Continuous variables are expressed as mean±standard error (SE), unless indicated differently. For group-wise comparisons, Mann–Whitney test or t-test were used as appropriate. All tests were performed 2-sided and a p<0.05 was considered as statistically significant.

## Results

### Global AS analysis by exon arrays

In a previous work [31], we analyzed gene expression differences in *biceps brachii* muscle biopsies of 10 DM2 and 10 control age and sex matched subjects (Table S2) using GeneChip Human Exon 1.0 ST Arrays. In this follow-up investigation, the same dataset was used to investigate AS events in genes exhibiting no difference in expression (Fig. 1). The Exon Array Analyzer (EAA) software analysis predicted 273 AS events distributed on 218 genes, with SI >+0.5 or <-0.5 and p-value <0.05 (Table S3). In particular, 164 splicing events had a positive SI and were classified as exon inclusions (SI>+0.5), while 54 had a negative SI and were exon deletions (SI<-0.5). Interestingly, 46 AS splicing events were already annotated in AStalavista (http://genome.crg.es/astalavista/) or in the UCSC Genome Browser (http://genome.ucsc.edu/), albeit most of them not in this specific disease context: 27 were cassette exons (both exon inclusion or exclusion), 6 were intron retentions, 10 were bleeding exons (initial or terminal exons overlap with an intron), while 3 events were not classified as AS, but as alternative promoter usage [39].

### Comparison with previous studies and qPCR validation

Numerous AS events identified by our screening had previously described as deregulated either in DM1 or DM2 or both (Table S4). For instance, as previously reported [17], the EAA software analysis identified MBNL1 (Muscleblind-like splicing regulator 1) exon 7 inclusion (Fig. S1) and FHOD1 (Formin HOmology 2 Domain containing 1) exon 11 inclusion (Fig. S2) events as more frequent in DM2 patients compared to controls. Furthermore, in keeping with previous data [21], [26], LDB3 (LIM Domain Binding 3, Fig. S3) and NFIX (Nuclear Factor I/X, Fig. S4) exhibited inclusion of exon 4 and exon 7, respectively, in DM2 patients. The preferential skipping in DM2 patients of exon 6 of MAPT/TAU (Microtubule-associated Protein Tau) was also confirmed (Fig. S5) [27], [40].

Finally, a very recent global analysis identified aberrant AS events in DM1 and DM2 [27]. Among those validated by reverse transcription PCR in a DM1 group, EAA software identified 28 AS events that were similarly deregulated in our DM2 cohort. Chi-square test confirmed that this overlap was statistically significant (p<0.001).

In order to further corroborate our analysis, we validated a subset of AS events by qPCR. To this aim, we used *biceps brachii* biopsies derived from 19 DM2 and 15 CTR age and sex matched patients. Of note, only 4 DM2 and 6 control individuals were in common between the screening and the validation cohorts (Tab S2 and S5).

**Figure 2:**
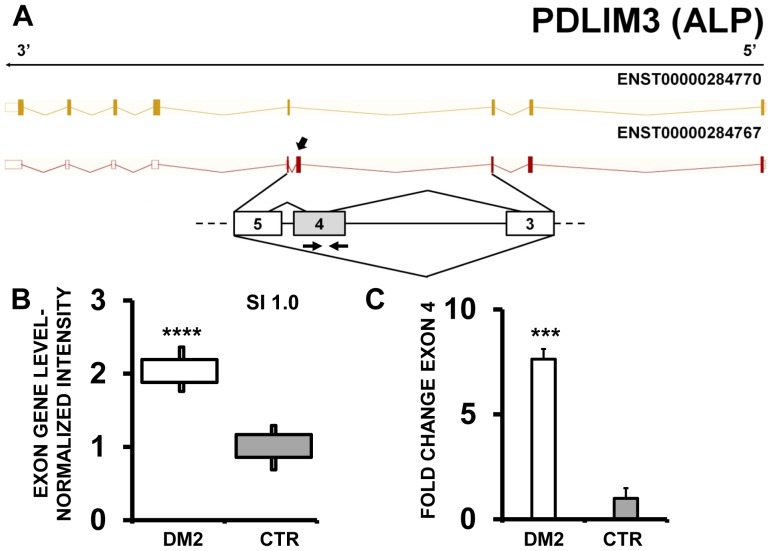
Increased PDLIM3 exon 4 inclusion in DM2 patients. A) EAA analysis identified an AS event on exon 4 of PDLIM3 mRNA transcript ENST00000284767. The AS area is enlarged and exon 4, more frequently included in DM2 patients, is highlighted in solid gray. B) The box plot shows the increased expression of Affymetrix probe set 2796971 recognizing exon 4, in DM2 patients compared to CTR (n = 10, **** p<0.0001). Values are normalized for the levels of the whole transcript. The splice index (SI) is indicated. C) Validation qPCR assays were performed using the specific primer pair indicated as black arrowhead in panel A. Results are shown as fold change (DM2 = 19, CTR = 15; *** p<0.001).

**Figure 3:**
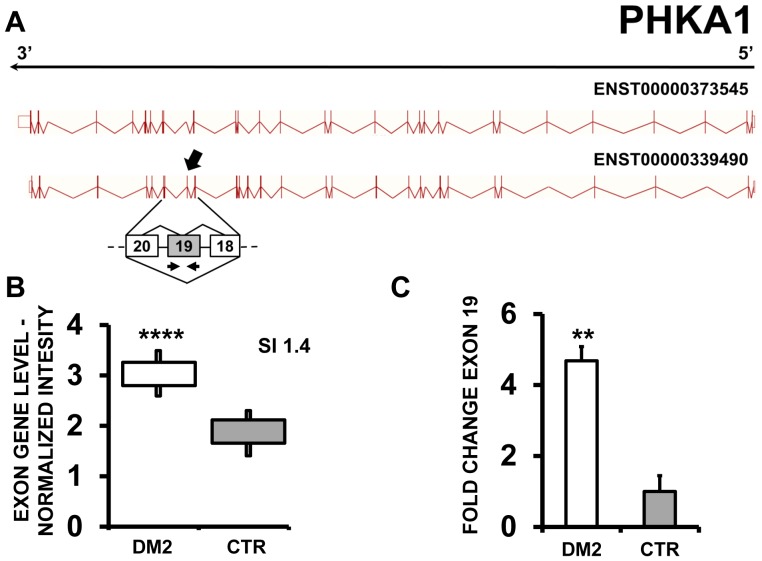
Increased PHKA1 exon 19 inclusion in DM2 patients. A) EAA analysis identified an AS event on exon 19 of PHKA1 mRNA transcript ENST00000339490. The AS area is enlarged and exon 19, more frequently included in DM2 patients, is highlighted in solid gray. B) The box plot shows the increased expression of Affymetrix probe set 4012322 recognizing exon 19, in DM2 patients compared to CTR (n = 10, **** p<0.0001). Values are normalized for the levels of the whole transcript. The splice index (SI) is indicated. C) Validation qPCR assays were performed using the specific primer pair indicated as black arrowhead in panel A. Results are shown as fold change (DM2 = 19, CTR = 15; ** p<0.01).

In keeping with previous reports [27], [41], EAA indicated that PDLIM3 (PDZ and LIM domain 3) (Fig.2A–B) and PHKA1 [phosphorylase kinase, alpha 1 (muscle)] (Fig.3 A–B) both displayed an exon cassette inclusion event in DM2 patients. We designed primers specific for exon 4 of PDLIM3 and exon 19 of PHKA1 (Tab. S1) and amplified the cDNAs. As expected, we found increased exon inclusion in DM2 patients for both genes (Fig. 2C and 3C).

**Figure 4:**
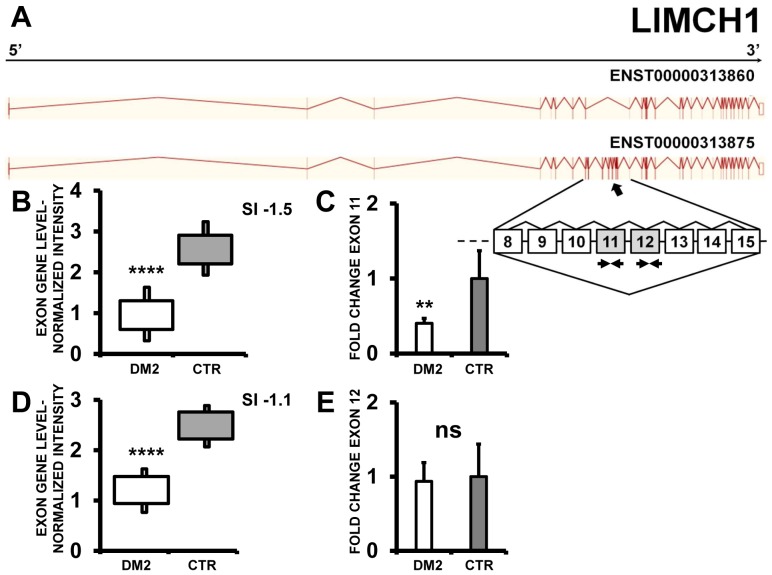
Increased LIMCH1 exon 11 skipping in DM2 patients. A) EAA analysis identified an AS event on exons 11 and 12 of LIMCH1 mRNA transcript ENST00000313875. The AS area is enlarged and exons 11 and 12, less frequently included in DM2 patients, are highlighted in solid gray. B and D) The box plots show the decreased expression of Affymetrix probe set 2725148 recognizing exon 11 (B) and 2725151 recognizing exon 12 (D), in DM2 patients compared to CTR (n = 10, **** p<0.0001). Values are normalized for the levels of the whole transcript. The splice index (SI) is indicated. C and E) Validation qPCR assays were performed using the specific primer pairs indicated as black arrowhead in panel A. Results are shown as fold change (DM2 = 19, CTR = 15; ** p<0.01). Only LIMCH1 exon 11 skipping was validated.

**Figure 5:**
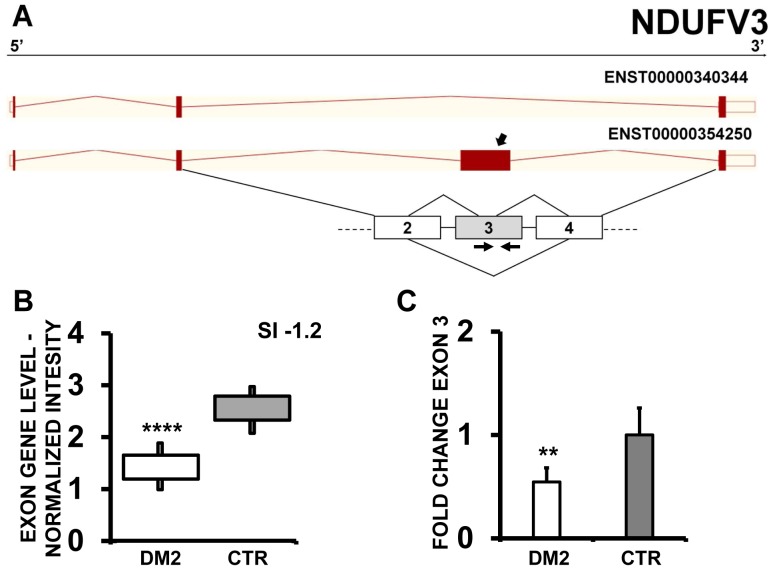
Increased NDUFV3 exon 3 skipping in DM2 patients. A) EAA analysis identified an AS event on exon 3 of NDUFV3 mRNA transcript ENST00000354250. The AS area is enlarged and exon 3, less frequently included in DM2 patients, is highlighted in solid gray. B) The box plot shows the decreased expression of Affymetrix probe set 3922937 recognizing exon 3, in DM2 patients compared to CTR (n = 10, **** p<0.0001). Values are normalized for the levels of the whole transcript. The splice index (SI) is indicated. C) Validation qPCR assays were performed using the specific primer pair indicated as black arrowhead in panel A. Results are shown as fold change (DM2 = 19, CTR = 15; ** p<0.01).

Likewise, two other genes among those described in both DM1 and DM2 [27], [41] were identified by EAA, LIMCH1 (LIM and calponin homology domains 1) and NDUFV3 (NADH Dehydrogenase (Ubiquinone) Flavoprotein 3), that both showed a exon cassette skipping event in DM2 compared to CTR. Specifically, exon 10 and 11 of LIMCH1 were predicted as significantly mis-spliced by EAA results (Fig.4). qPCR experiments confirmed the more frequent inclusion of exon 10. However, differences in exon 11 inclusion in DM2 patients did not reach statistical significance (Fig.4E). In the case of NDUFV3, EAA outputs showed exon 3 skipping in DM2 patients compared to CTR (Fig. 5A and B); these data were confirmed by qPCR using a exon 3-specific primer pair (Fig. 5C).

**Figure 6:**
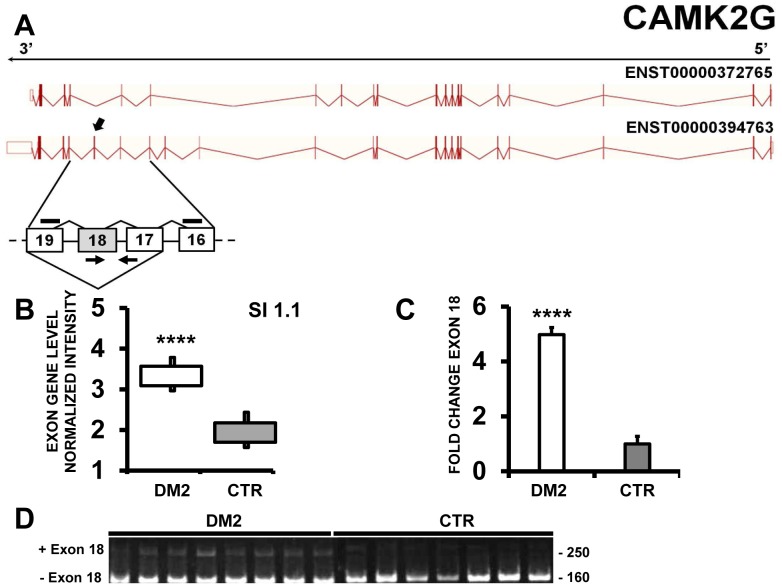
Increased CAMK2G exon 18 inclusion in DM2 patients. A) EAA analysis identified an AS event on exon 18 of CAMK2G mRNA transcript ENST00000394763. The AS area is enlarged and exon 18, more frequently included in DM2 patients, is highlighted in solid gray. B) The box plot shows the increased expression of Affymetrix probe set 3294875 recognizing exon 18, in DM2 patients compared to CTR (n = 10, **** p<0.0001). Values are normalized for the levels of the whole transcript. The splice index (SI) is indicated. C) Validation qPCR assays were performed using the specific primer pair indicated as black arrowhead in panel A. Results are shown as fold change (DM2 = 19, CTR = 15; **** p<0.0001). D) Reverse transcription PCR products obtained with the specific primer pair indicated as black lines in panel A, were resolved on 5% polyacrylamide electrophoresis gel. The 250 bp fragment containing exon 18 was more abundant in DM2 patients.

Nakamori et al. identified in DM1 patients the retention of 18–19 intron in CAMK2G (calcium/calmodulin-dependent protein kinase II gamma) transcript [27], [41]. EAA analysis of our data did not detect this event, but indicated that exon 18 of CAMK2G was more frequently retained in DM2 compared to CTR samples (Fig.6B). This result was confirmed by qPCR using a primer pair spanning from exon 17 to exon 18 (Fig.6C). Moreover, using primers spanning from exon 16 to exon 19, the obtained amplicon was resolved on 5% PAGE (Fig.6D). As expected, both DM2 and CTR displayed a 160 bp amplicon corresponding to the transcript skipping exon 18. However, DM2 patients also showed a 248 bp amplicon associated to an exon 18-including transcript. The identity of the amplicons and the fidelity of the exon 17/18 and 18/19 junctions were confirmed by DNA sequencing.

Next, we extended our analysis to other AS events not previously associated to DM1 or DM2, testing a variety of AS events.

**Figure 7:**
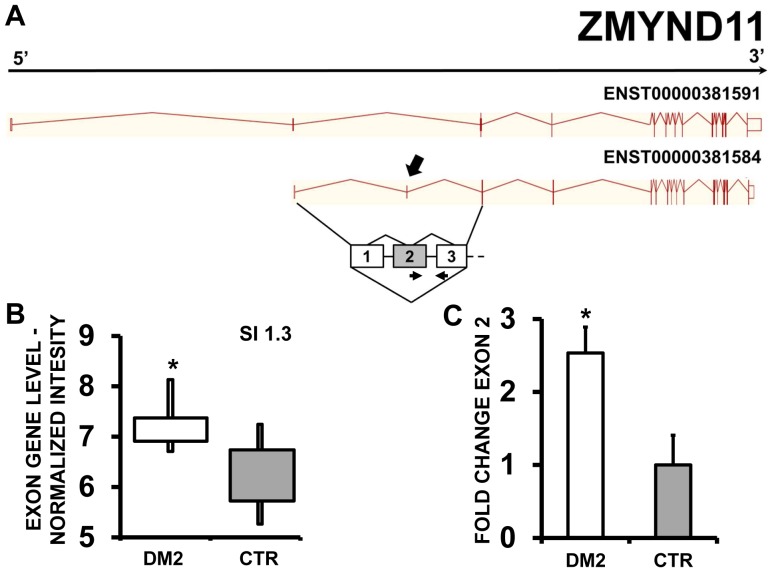
Increased ZMND11 exon 2 inclusion in DM2 patients. A) EAA analysis identified an AS event on exon 2 of ZMND11 mRNA transcript ENST00000381584. The AS area is enlarged and exon 2, more frequently included in DM2 patients, is highlighted in solid gray. B) The box plot shows the increased expression of Affymetrix probe set 3231406 recognizing exon 2, in DM2 patients compared to CTR (n = 10, * p<0.05). Values are normalized for the levels of the whole transcript. The splice index (SI) is indicated. C) Validation qPCR assays were performed using the specific primer pair indicated as black arrowhead in panel A. Results are shown as fold change (DM2 = 19, CTR = 15; * p<0.05).

**Figure 8:**
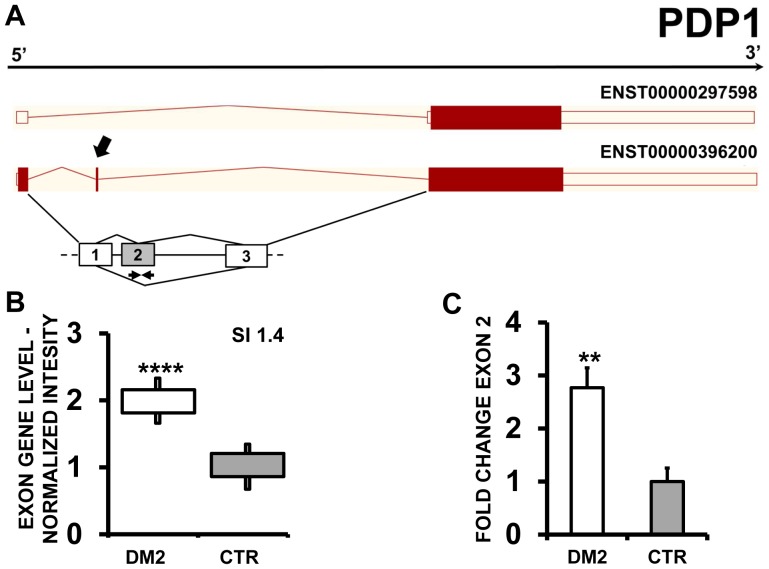
Increased PDP1 exon 2 inclusion in DM2 patients. A) EAA analysis identified an AS event on exon 2 of PDP1 mRNA transcript ENST00000396200. The AS area is enlarged and exon 2, more frequently included in DM2 patients, is highlighted in solid gray. B) The box plot shows the increased expression of Affymetrix probe set 3107346 recognizing exon 2, in DM2 patients compared to CTR (n = 10, **** p<0.0001). Values are normalized for the levels of the whole transcript. The splice index (SI) is indicated. C) Validation qPCR assays were performed using the specific primer pair indicated as black arrowhead in panel A. Results are shown as fold change (DM2 = 19, CTR = 15; ** p<0.01).

**Figure 9:**
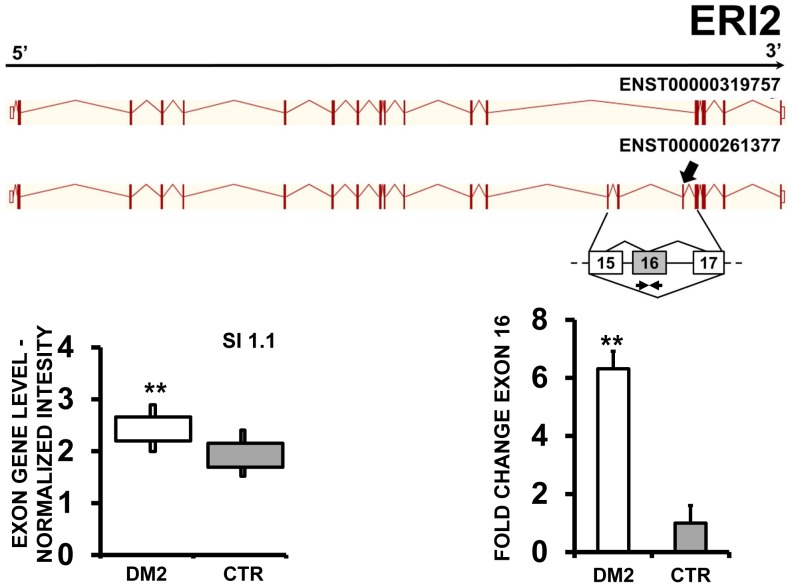
Increased ERI2 exon 16 inclusion in DM2 patients. A) EAA analysis identified an AS event on exon 16 of ERI2 mRNA transcript ENST00000261377. The AS area is enlarged and exon 16, more frequently included in DM2 patients, is highlighted in solid gray. B) The box plot shows the increased expression of Affymetrix probe set 3651550 recognizing exon 16, in DM2 patients compared to CTR (n = 10, ** p<0.01). Values are normalized for the levels of the whole transcript. The splice index (SI) is indicated. C) Validation qPCR assays were performed using the specific primer pair indicated as black arrowhead in panel A. Results are shown as fold change (DM2 = 19, CTR = 15; ** p<0.01).

**Figure 10:**
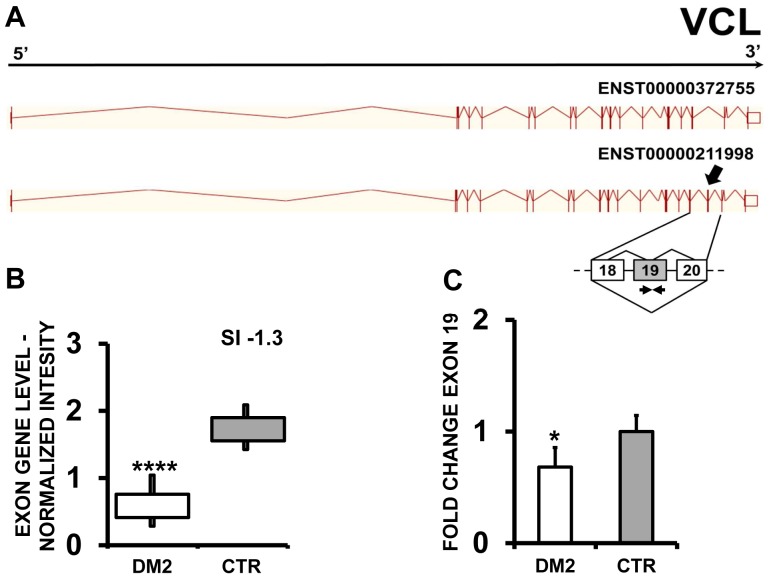
Increased VCL exon 19 skipping in DM2 patients. A) EAA analysis identified an AS event on exon 19 of VCL mRNA transcript ENST00000211998. The AS area is enlarged and exon 19, more frequently excluded in DM2 patients, is highlighted in solid gray. B) The box plot shows the decreased expression of Affymetrix probe set 3252129 recognizing exon 19, in DM2 patients compared to CTR (n = 10, **** p<0.0001). Values are normalized for the levels of the whole transcript. The splice index (SI) is indicated. C) Validation qPCR assays were performed using the specific primer pair indicated as black arrowhead in panel A. Results are shown as fold change (DM2 = 19, CTR = 15; * p<0.05).

EAA results indicated that ZMYND11 (zinc finger, MYND-type containing 11) (Fig. 7 A and B), PDP1 (pyruvate dehyrogenase phosphatase catalytic subunit 1) (Fig. 8 A and B) and ERI2 (ERI1 exoribonuclease family member 2) (Fig. 9 A and B) displayed an exon cassette inclusion event, while VCL (vinculin) (Fig. 10 A and B) displayed and exon cassette skipping event. We designed primers specific for exon 2 of ZMYND11, exon 2 of PDP1, exon 16 of ERI2 and exon 19 of VCL (Tab S1) and amplified the cDNAs. qPCR confirmed all predicted AS events (Figs. 8C, 9C and 10 C).

**Figure 11:**
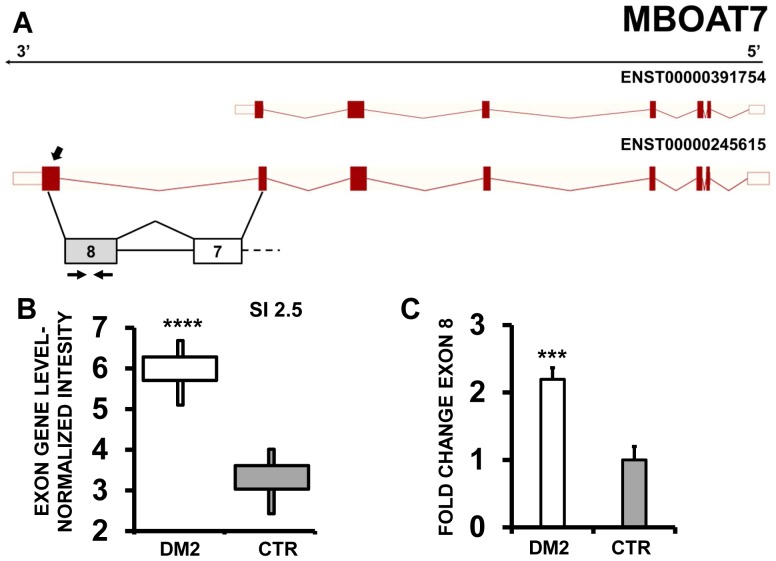
Increased MBOAT7 exon 8 inclusion in DM2 patients. A) EAA analysis identified an AS event on exon 8 of MBOAT7 mRNA transcript ENST00000245615. The AS area is enlarged and exon 8, more frequently included in DM2 patients, is highlighted in solid gray. B) The box plot shows the increased expression of Affymetrix probe set 3870571, recognizing exon 8 in DM2 patients compared to CTR (n = 10; **** p<0.0001). Values were normalized for the levels of the whole transcript. The splice index (SI) is indicated. C) Validation qPCR assays were performed using the specific primer pair indicated as black arrowhead in panel A. Results are shown as fold change (DM2 = 19, CTR = 15; *** p<0.001).

**Figure 12:**
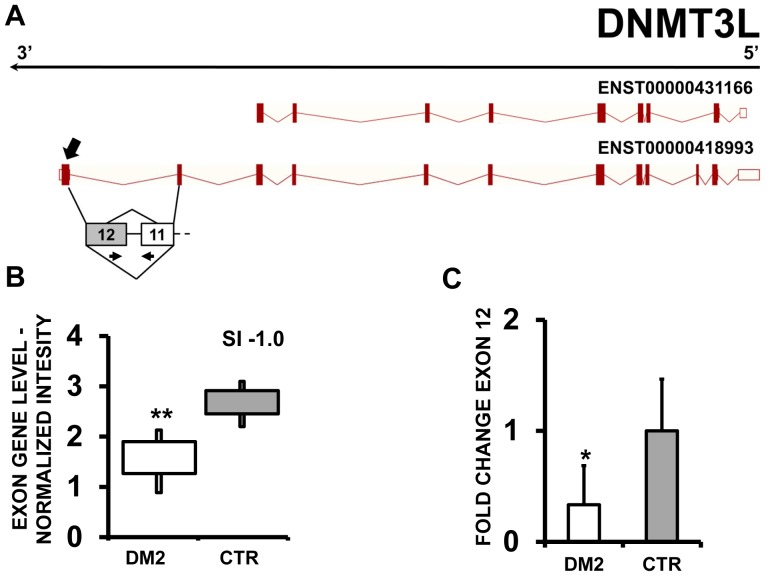
Increased DNMT3L exon 12 skipping in DM2 patients. A) EAA analysis identified an AS event on exon 12 of DNMT3L mRNA transcript ENST00000418993. The AS area is enlarged and exon 12, more frequently excluded in DM2 patients, is highlighted in solid gray. B) The box plot shows the decreased expression of Affymetrix probe set 3934442 recognizing exon 12, in DM2 patients compared to CTR (n = 10, ** p<0.01). Values are normalized for the levels of the whole transcript. The splice index (SI) is indicated. C) Validation qPCR assays were performed using the specific primer pair indicated as black arrowhead in panel A. Results are shown as fold change (DM2 = 19, CTR = 15; ** p<0.01).

**Figure 13:**
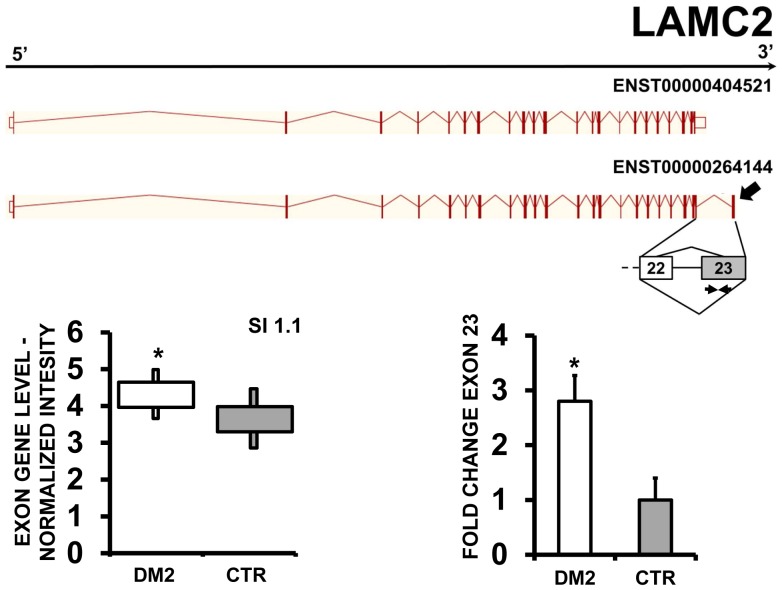
Increased LAMC2 exon 23 inclusion in DM2 patients. A) EAA analysis identified an AS event on exon 23 of LAMC2 mRNA transcript ENST00000264144. The AS area is enlarged and exon 23, more frequently included in DM2 patients, is highlighted in solid gray. B) The box plot shows the increased expression of Affymetrix probe set 2371184, recognizing exon 23, in DM2 patients compared to CTR (n = 10, * p<0.05). Values are normalized for the levels of the whole transcript. The splice index (SI) is indicated. C) Validation qPCR assays were performed using the specific primer pair indicated as black arrowhead in panel A. Results are shown as fold change (DM2 = 19, CTR = 15; *p<0.05).

Moreover, EAA indicated that MBOAT7 (Membrane Bound O-AcylTransferase domain containing 7) (Fig. 11 A–B), DNMT3L (DNMT3L DNA (cytosine-5-)-methyltransferase 3-like) (Fig. 12 A–B) and LAMC2 (laminin, gamma 2) (fig.13 A–B) displayed alternative inclusion of the last exon. We designed primers specific for exon 8 of MBOAT7, exon 12 of DNMT3L and exon 23 of LAMC2 and amplified the cDNAs. qPCR confirmed all AS events (Figs 11 C,12C and 13C).

**Figure 14:**
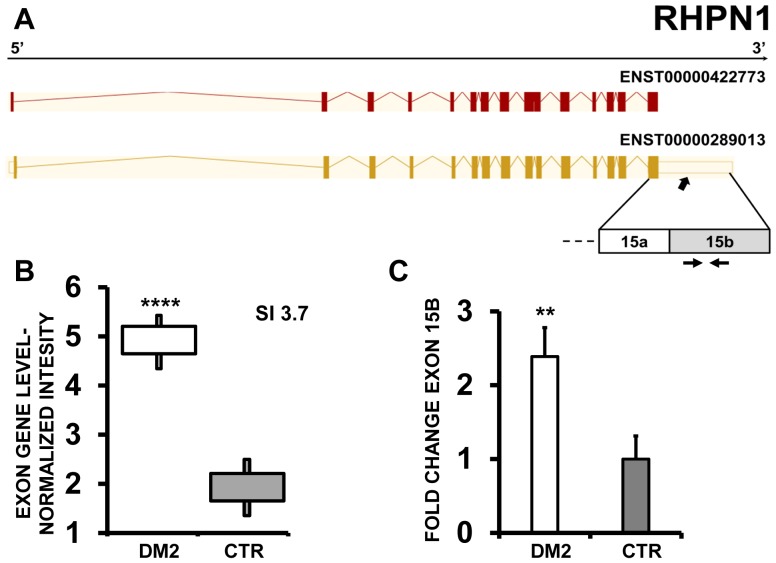
Increased RHPN1 exon 15 bleeding in DM2 patients. A) EAA analysis identified an AS event on exon 15b of RHPN1 mRNA transcript ENST00000289013. The AS area is enlarged and exon 15b, more frequently included in DM2 patients, is highlighted in solid gray. B) The box plot shows the increased expression of Affymetrix probe set 3119603 recognizing exon 15b in DM2 patients compared to CTR (n = 10, **** p<0.0001). Values were normalized for the levels of the whole transcript. The splice index (SI) is indicated. C) Validation qPCR assays were performed using the specific primer pair indicated as black arrowhead in panel A. Results are shown as fold change (DM2 = 19, CTR = 15; *** p<0.001).

In a further experiment, RHPN1 (Rhophilin, Rho GTPase binding protein 1) showed a higher expression of bleeding exon 15b in DM2 compared to CTR (Fig. 14A, B). Indeed, qPCR experiments confirmed the more frequent inclusion of exon 15b in DM2 patients (Fig. 14 C).

**Figure 15:**
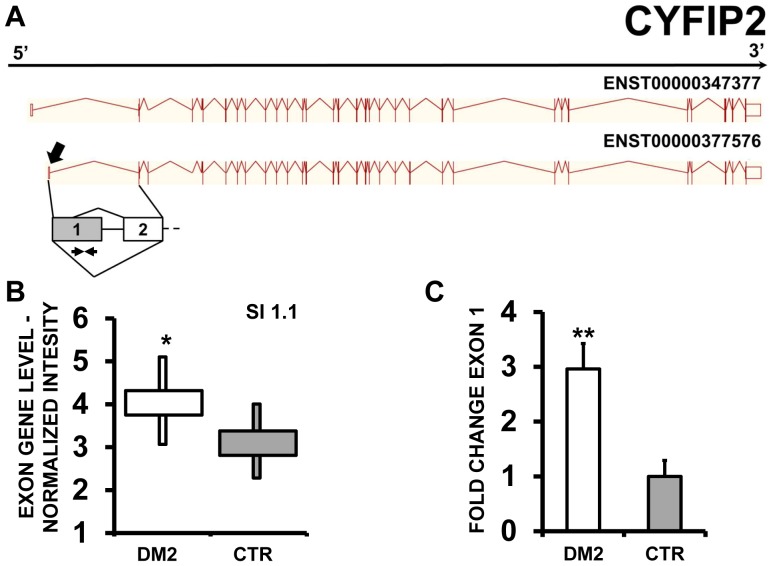
Increased CFYIP2 exon 1 inclusion in DM2 patients. A) EAA analysis identified an AS event on exon 1 of CFYIP2 mRNA transcript ENST00000377576. The AS area is enlarged and exon 1, more frequently included in DM2 patients, is highlighted in solid gray. B) The box plot shows the increased expression of Affymetrix probe set 2837276 recognizing exon 1, in DM2 patients compared to CTR (n = 10, * p<0.05). Values are normalized for the levels of the whole transcript. The splice index (SI) is indicated. C) Validation qPCR assays were performed using the specific primer pair indicated as black arrowhead in panel A. Results are shown as fold change (DM2 = 19, CTR = 15; ** p<0.01).

Finally, CFYIP (cytoplasmic FMR1 interacting protein 2) mRNA transcripts presented an alternative transcript start, with a prominent expression of the shorter isoform in DM2 patients compared to CTR subjects (Fig. 15 A and B). Again, qPCR assays using specific primer pairs confirmed this event (Fig. 15 C).

### Pathways and Networks of genes with aberrant splicing events

To gain insight into the molecular pathways involving the identified aberrantly spliced genes, Interactive Pathway Analysis (IPA) of experimental data was performed by Ingenuity software. Using the list of 218 genes involved in AS events predicted by EAA, IPA identified several pathways and functions that might be relevant for DM2. Top categories are shown on Tab.1, while the complete list of significant categories is represented in Tab. S6. Particularly worth noting are “Skeletal and Muscular Disorders” and “Neurological Diseases” categories among Diseases, “Cell Death and Survival” and “Cellular Development” among Molecular and Cellular Functions, “Calcium signaling” among Pathways and “Cardiac Arrythmia” among Cardiac Toxic Functions.

**Table 1:** Most significant categories and functions.

DISEASE AND DISORDERS	p-value
Immunological disease	3.11E-04 -2.13E-02
Neurological disease	3.11E-04 -2.30E-02
Skeletal and Muscular Disorders	3.11E-04 -1.77E-02
Cancer	9.22E-04 -2.46E-02
Reproductive System Disease	9.22E-04 -1.77E-02
**MOLECULAR AND CELLULAR FUNCTIONS**	**p-value**
Cell Death and Survival	1.05E-04 -2.13E-02
Cellular Development	1.81E-04 -1.83E-02
Cell Morphology	2.85E-04 -1.77E-02
Cellular Movement	5.95E-04 -2.46E-02
Cell-To-Cell Signaling and Interaction	1.25E-03 -2.46E-02
**PATHWAYS**	**-log(p-value)**
Lanosterol Biosynthesis	1.75E00
Netrin Signaling	1.73E00
Epithelial Adherens Junction Signaling	1.62E00
Fatty Acid Biosynthesis Initiation II	1.46E00
Palmitate Biosynthesis I (Animals)	1.46E00
Urea Cycle	1.46E00
Calcium Signaling	1.42E00
**TOP CARDIOTOXIC FUNCTIONS**	**p-value**
Increased Levels of Albumin	1.77E-02 -1.77E-02
Increased Levels of Alkaline Phosphatase	1.77E-02 -6.12E-01
Cardiac Arrythmia	4.07E-03 -3.25E-01
Tachycardia	4.07E-03 -3.25E-01
Cardiac Dilation	1.77E-02 -1.45E-01
Congenital Heart Anomaly	1.77E-02 -4.35E-01
Cardiac Hypoplasia	3.95E-02 -3.95E-02

Then, using MetaCore integrated software, we analyzed the networks produced by the list of recognized AS events. It was analyzed by using the “Direct interactions algorithm”, which creates a network only from the objects under analysis (Fig. S6). In the core of this network, we could find genes like c-Abl, VCL, DNA-PK, c-Src, beta-catenin, and WNK. Interestingly the last 4 genes were also found within the DMPK (Dystrophia Myotonica Protein Kinase) gene network (See Methods) (Fig. S7).

Expanding automatically our dataset using the ‘Analyze Networks’ algorithm (see Methods), amongst the first enriched subnetworks, we found one containing CDC42BPB (CDC42 binding protein kinase beta (DMPK-like)) with DNA-PK and SHP2 in the core (Fig. S8) and another with IDH3 (Isocitrate DeHydrogenease 3) with respiratory complex III and c-Myc in its core and containing RHPN1 (Fig. S9). Indeed, when networks for DM-related genes were built (see Methods) an intersection between our dataset was found in the CDC42BPB subnetwork consisting in CDC42BPB, MRCK and PPRC (not shown).

## Discussion

Dysregulation of AS is a fundamental molecular trait of DM2, affecting many genes involved in muscle homeostasis and function [18], [20]–[23], [25]–[27], [42]–[44]. Thus, the identification of the AS alterations is a crucial step for our understanding of the pathogenetic mechanisms of the disease and for the identification of biomarkers of functional impairment [27]. However, gaining insight in the molecular network of the gene expression and AS alterations triggered by the DM2 genetic lesion is hurdled by the variability between patients and between different muscles of the same subject [27]. Another substantial obstacle is constituted by the fact that DM2 is a rare disease and by the difficulty of obtaining specimens with intact RNA. The current study validates many known DM2-affected splice events and further expands its number. Indeed, we identified in our profiling group 35 AS alterations previously described in DM1, DM2 or both. In order to minimize the number of false positives, we adopted very stringent inclusion criteria. As a consequence, we can postulate the presence of some false negatives. Accordingly, adopting more inclusive criteria (with IterPLIER gene and exon level normalization) several other known AS events were also present in our dataset, such as SOS1 exon 25 skipping [27], BIN1 exon 11 inclusion [42], OPA1 exon 4 skipping [27], VEGFA exon 6 inclusion [27], MBNL2 exon 7 inclusion [27], ALPK3 exon 2 inclusion [27], PHK1 exon 19 inclusion [27]. Moreover, exon 22 skipping of ATP2A1 [25] displayed a SI value that was just below threshold, but was consistently validated by PCR (AP, SG and FM, unpublished). Finally, certain genes displayed very low or no detectable signals in our array analysis. Thus, data on well characterized AS aberrations, such as INSR exon 11 skipping [20], [45] or ClC1 intron 2 inclusion [18], [45]–[47] were inconclusive.

Intriguingly, we also found that exon inclusion AS events were more common than exon skipping ones. While this may be due to technical reasons, it is worth noting that a similar trend was also observed in other studies [21], [27], [28].

The emerging scenario shows that we still are in a first phase of data acquisition that needs to be followed by an accurate meta-analysis in order to dissect out the molecular pathways and the single genes leading to disease form simple “bystanders”. In spite of this mandatory cautionary note, we can hypothesize that the identified AS events are likely to be of pathogenetic importance. Indeed, IPA analysis of pathways and functions involving the identified genes displayed several relevant top-scoring hits. Significantly, this analysis indicated that among Diseases, “Neurological Diseases” and “Skeletal and Muscular Disorders” were the second and the third top categories, respectively.

Furthermore, among relevant gene networks identified there were those involving IDH3, DMPK and DMPK-like CDC42BPB. The IDH3isozyme is a heterotetramer mitochondrial enzyme that is composed of two alpha subunits (IDH3A), one beta subunit (IDH3B), and one gamma subunit (IDH3G) [48], [49]. IDH3B and G isoforms are both aberrantly spliced in DM2 patients and this deregulation correlated with the mitochondrial pathways dysfunction observed in DM2 muscles [50].

The DMPK network is also of obvious relevance, given its role in DM1 pathogenesis [2]–[4]. Unlike CTG triple expansion, DMPK deficiency does not seem to play an essential role in the pathogenetic mechanism. Accordingly, Dmpk−/− mice do not reproduce the complex and multisystemic DM1 phenotype, suggesting that haploinsufficiency of this gene is not the primary mechanism of disease. Nevertheless, DMPK inactivation might contribute to altered ion homeostasis in muscle and heart. [51], [52]. Moreover, DMPK promotes myogenic gene expression in skeletal myoblasts and its disruption may contribute to skeletal muscle wasting [53]. Thus, our finding indicates that the aberrant splicing of DMPK network component c-Src, WNK1, DNA-PK, beta-catenin and TYRP1 may be functionally relevant.

Also interesting is the AS deregulation of CDC42BPB and of other components of its network, given that CDC42BPB is a kinase protein that is involved in cytoskeletal reorganization and actin formation [54].

A subset of identified AS was also validated by PCR in a larger patient cohort. Our investigation and the one of Nakamori et al.[27] identified several AS events in the LIMCH1 gene in DM2 and DM1, respectively. Both studies failed to confirm exon 12 skipping both in DM1 [27] and DM2 (this study), underlining the importance of data validation with an independent technique. However, we successfully validated exon 11 skipping, confirming that LIMCH1 is a target of DM2 AS aberrations.

Array analysis also identified a more frequent inclusion of PDLIM3 exon 4 in DM2 patients and this event was validated by qPCR. Our data are in agreement with the observations of Ohsawa and collaborators in DM1 patients [41]. They found that the isoforms predominant in CTR are PDLIM3a (exon 5/6 inclusion/exon 4 exclusion isoform) and PDLIM3c (exon 5 inclusion and exons 4/6 exclusion isoform), while DM1 muscles expressed the PDLIM3b isoform (exon 4 inclusion and exons 5/ 6 exclusion isoform). Lin et al. [17] also found in both DM1and DM2 muscles that PDLIM3 exon 5 was alternatively spliced as well. However, in our DM2 patient group, EAA data did not predict significant AS events in exons other than exon 4. Of note, AS of LIMCH1 exon 4 was also observed in the hippocampus of Mbnl2 -/- mice, suggesting that this event might under MBNL control also in human skeletal muscles [55].

The PDLIM3b isoform, with exon 4 inclusion, is mainly expressed in fetal skeletal muscles, whereas PDLIM3a and PDLIM3c are predominantly detected after birth [41]. Thus, the predominant expression of the fetal exon 4 in DM2 muscle is consistent with the reactivation of a fetal gene program due to the muscle disease [56], [57]. Moreover, PDLIM3 seems to have a role in muscle differentiation since the knocking down of PDLIM3 affects the expression of myogenin and MyoD [58]. Furthermore, while PDLIM3a and PDLIM3c are expressed predominantly in muscle (heart and skeletal muscles), PDLIM3b has a more ubiquitous expression pattern, suggesting that exon 4 containing isoforms may have functions other than those executed in skeletal muscles [41]. It is also worth noting that three aberrantly spliced genes, PDLIM3, PDLIM5 and LDB3, all belong to the same family [59],suggesting the impairment of a whole molecular function.

Another protein family that is affected by DM2 AS disruption is CAMK2. Indeed, we found that CAMK2B and CAMK2G are aberrantly spliced in DM2 patients. Accordingly, Nakamori et al. recently showed that the AS of CAMK2A and CAMK2G is affected in DM1 too, although for CAMK2G a different AS event was observed. The aberrant AS of CAMK2 genes are of particular interest since several studies indicated that Ca2+ signaling is probably the most deregulated pathway in both DM patients and mouse models [17], [21], [22], [25], [26], [44], [60], [61]. Accordingly, in our IPA analysis, “Calcium signaling” is among the top affected Pathways. Moreover, Nakamori et al [27] found that splicing of CAMK2G was affected also in Mbnl1 knockout mice, suggesting that Mbnl1 sequestration might influence the AS of this gene.

CAMK2 is a multimeric ubiquitous holoenzyme regulating many pathways in response to Ca2+ signaling, including neurotransmitter synthesis and release, cytoskeleton organization and calcium homeostasis [62]–[64]. Expressed from 4 CaMK2 genes (A, B, G and D or α, β, γ and δ), over 20 different types of CaMK2 have been identified [65], [66]. The primary difference between the CaMK2 isoforms results from a series of inserts named “variable regions” laying between the Ca2+/CaM-binding domain and the association domain [64]. These variable regions modulate substrate specificity, calmodulin regulation, holoenzyme formation, or subcellular, targeting/localization [67]. Of relevance, the aminoacids encoded by the exons aberrantly spliced in CAMK2G (exon 18), CAMK2B (exon 16/17) and CAMK2A (exon 14) all lay in the variable regions [64].

In another study, it has been shown that the presence of variable region I in CaMKIIG lowered the amount of Ca2+/calmodulin required for activation [68]. Thus, alterations of the variable regions of the CAMK2 family may lead to an increased binding affinity of the protein to the Ca2+/CaM complex that, in turn, ameliorates the muscle weakness characterizing the disease. On the other hand, a tighter CAMK2 to CaM interaction may also perturb the graduation of the responses following repeated Ca2+ spikes in the cytoplasm.

In conclusion, our genome wide analysis provided a database of aberrant splicing events in the skeletal muscle of DM2 patients. The affected genes are involved in numerous pathways and networks important for muscle physio-pathology, suggesting that the identified variants may contribute to DM2 pathogenesis.

## Supporting Information

Figure S1 Increased MBNL1 exon 7 inclusion in DM2 patients. A) EAA analysis identified an AS event on exon 7 of MBNL1 mRNA transcript. ENST00000282486. The AS area is enlarged and exon 7, more frequently included in DM2 patients, is highlighted in solid gray. B) The box plot shows the increased expression of Affymetrix probe set 2648174 recognizing exon 7, in DM2 patients compared to CTR (n = 10, **** p<0.0001). Values are normalized for the levels of the whole transcript. The splice index (SI) is indicated.
(PPTX)

Figure S2 Increased FHOD1 exon 11 inclusion in DM2 patients. A) EAA analysis identified an AS event on exon 11 of FHOD1 mRNA transcript ENST00000258201. The AS area is enlarged and exon 11, more frequently included in DM2 patients, is highlighted in solid gray. B) The box plot shows the increased expression of Affymetrix probe set 3695543 recognizing exon 11 in DM2 patients compared to CTR (n = 10, *** p<0.001). Values are normalized for the levels of the whole transcript. The splice index (SI) is indicated.
(PPTX)

Figure S3 Increased LDB3 exon 4 inclusion in DM2 patients. A) EAA analysis identified an AS event on exon 4 of LDB3 mRNA transcript ENST00000372056. The AS area is enlarged and exon 4, more frequently included in DM2 patients, is highlighted in solid gray. B) The box plot shows the increased expression of Affymetrix probe set 3255989 recognizing exon 4, in DM2 patients compared to CTR (n = 10, **** p<0.0001), as found by Vihola et al., 2010. Values are normalized for the levels of the whole transcript. The splice index (SI) is indicated.
(PPTX)

Figure S4 Increased NFIX exon 7 inclusion in DM2 patients. A) EAA analysis identified an AS event on exon 7 of NFIX mRNA transcript ENST00000358552. The AS area is enlarged and exon 7, more frequently included in DM2 patients, is highlighted in solid gray. B) The box plot shows the increased expression of Affymetrix probe set 3822162 recognizing exon 7, in DM2 patients compared to CTR (n = 10, **** p<0.0001). Values are normalized for the levels of the whole transcript. The splice index (SI) is indicated.
(PPTX)

Figure S5 Increased MAPT exon 6 skipping in DM2 patients. A) EAA analysis identified an AS event on exon 6 of MAPT mRNA transcript ENST00000344290. The AS area is enlarged and exon 6, less frequently included in DM2 patients, is highlighted in solid gray. B)The box plot shows the decreased expression of Affymetrix probe set 3723723 recognizing exon 6, in DM2 patients compared to CTR (n = 10, **** p<0.0001). Values are normalized for the levels of the whole transcript. The splice index (SI) is indicated.
(PPTX)

Figure S6 Direct Interactions Network of LIMMA significant genes. The algorithm created a network consisting only of genes with significant AS events and no other elements were added to the network. Only genes with one or more connections are shown. Up-regulated genes are marked with red circles; down-regulated with blue circles. Intensity of color corresponds to intensity of fold change.
(PPTX)

Figure S7 DMPK gene network. Network generated by the ‘Auto Expand’ algorithm around DMPK gene. The algorithm added genes to the network giving preference to those with more connectivity to the initial gene and halted when the sub-networks intersect or the overall size reached a pre-established limit of 50 genes. Genes in our data set, such as b-catenin, c-src, DNA-PK and WNK1 were found within the network. The genes with colored circles are those with AS significant events. Up-regulated genes are marked with red circles; down-regulated with blue circles. Intensity of color corresponds to intensity of fold change.
(PPTX)

Figure S8 CDC42BP network. Enriched sub network by ‘Analyze Network’ algorithm from LIMMA significant genes containing CDC42BP (DMPK-like) gene. The genes with colored circles are those with AS significant events. Up-regulated genes are marked with red circles; down-regulated with blue circles. Intensity of color corresponds to intensity of fold change.
(PPTX)

Figure S9 IDH3 network. Enriched sub network by ‘Analyze Network’ algorithm from LIMMA significant genes containing IDH3 genes. The genes with colored circles are those with AS significant events. Up-regulated genes are marked with red circles; down-regulated with blue circles. Intensity of color corresponds to intensity of fold change.
(PPTX)

Table S1 Primers sequences.
(XLSX)

Table S2 Clinical characteristics of the patients used for array profiling.
(XLSX)

Table S3 AS events predicted by EAA analysis.
(XLSX)

Table S4 Previously described DM-AS events predicted by EAA analysis.
(XLSX)

Table S5 Clinical characteristics of the patients used for PCR validation.
(XLSX)

Table S6 Complete enrichment analysis report.
(XLSX)
