# Correction: Genome Wide Identification of Aberrant Alternative Splicing Events in Myotonic Dystrophy Type 2

**DOI:** 10.1371/journal.pone.0108102

**Published:** 2014-09-19

**Authors:** The PLOS ONE Staff

The sixth author’s name is spelled incorrectly. The correct name is: Jose M. Garcia-Manteiga.
